# Bidirectional Crosstalk between Lymphatic Endothelial Cell and T Cell and Its Implications in Tumor Immunity

**DOI:** 10.3389/fimmu.2017.00083

**Published:** 2017-02-06

**Authors:** Kim Pin Yeo, Veronique Angeli

**Affiliations:** ^1^Immunology Programme, Department of Microbiology and Immunology, Yoon Loo Lin School of Medicine, Life Science Institute, National University of Singapore, Singapore, Singapore

**Keywords:** lymphatic endothelial cells, T cell, lymph node, cancer, inflammation, tolerance, cytokine

## Abstract

Lymphatic vessels have been traditionally considered as passive transporters of fluid and lipids. However, it is apparent from recent literature that the function of lymphatic vessels is not only restricted to fluid balance homeostasis but also extends to regulation of immune cell trafficking, antigen presentation, tolerance, and immunity, all which may impact the progression of inflammatory responses and diseases such as cancer. The lymphatic system and the immune system are intimately connected, and there is emergent evidence for a crosstalk between T cell and lymphatic endothelial cell (LEC). This review describes how LECs in lymph nodes can affect multiple functional properties of T cells and the impact of these LEC-driven effects on adaptive immunity and, conversely, how T cells can modulate LEC growth. The significance of such crosstalk between T cells and LECs in cancer will also be discussed.

## Lymph Node (LN) Architecture

Lymph nodes are strategically positioned and highly organized organs that serve as “rendez-vous” points for dendritic cells (DCs), T cells, and B cells. The maintenance of LN structure and compartmentalization are essential for the elicitation and development of effective immune response. LN can be subdivided into three main regions, namely, the cortex, the paracortex, and the medulla. Encapsulated LNs receive lymph from peripheral tissue and organs through the afferent lymphatic. Molecules, antigens, microorganisms, and cells such as lymphocytes and antigen-presenting cells (APCs) within the lymph are emptied into to the subcapsular sinus (SCS) of the LN. Subcapsular and medullary sinuses are directly interconnected, and hence, lymph-borne cells, fluid, and soluble molecules can pass through LN without percolating through the cortex (1). Within the SCS resides CD169-expressing macrophage and DC; these cells capture large molecules, particles, and microorganisms; and then display antigens to the lymphocytes (2–4). Densely packed B cells and follicular dendritic cells (FDCs) are organized into discrete B cell follicles in the cortex. FDCs cluster in the center of the follicles and form a dense network in which B cells contact with the antigens. Lymphocytes mainly enter LNs from the blood *via* high endothelial venules (HEVs) (5). T cell zones of the paracortex contain CD4^+^ and CD8^+^ T cells and subsets of DCs in close contact with a network of conduits formed by fibroblastic reticular cells (FRCs). The medulla is composed of a three-dimensional labyrinthine structure of sinus channels starting as cortical sinusoids and expands to become wider medullary sinuses that finally drain collectively into the efferent lymphatic vessel (6).

Lymph nodes consist of not only hematopoietic cells (CD45^+^) but also heterogeneous populations of non-hematopoietic cells (CD45^−^). Currently, there are five major stromal cell subsets that have been characterized, namely, the marginal reticular cells (MRCs), FRCs, lymphatic endothelial cells (LECs), blood endothelial cells (BECs), and FDCs. They can be identified by their anatomical location within the LN and by the expression of CD31, podoplanin (also known as Gp38), CD35 (complement receptor 1), and mucosal addressin cell adhesion molecule-1 (MadCAM-1). MRCs and FRCs express Gp38 but not CD35 and CD31. MRCs can be delineated from FRCs not only by their expression of MadCAM-1 but also by their localization in the outer follicular region immediately underneath the SCS (7). LECs express both CD31 and Gp38, whereas BECs express only CD31. FDCs are centrally located within B cell follicles and are often classified based on the expression of CD21/CD35 (8), FDC-M1 (9), and FDC-M2 (complement C4) (10). Conventionally, stromal cells have long been perceived to provide structural support to the LNs during homeostasis and inflammation. Emerging evidence also indicates that stromal compartments of LNs play active roles in the immune response through their interactions with hematopoietic cells. We will briefly discuss here the role of FRCs as it has been covered recently in excellent reviews (11–13), and this review focuses on LECs.

## Fibroblastic Reticular Cells

Fibroblastic reticular cells are resident mesenchymal cells, primarily residing in the T cells zone and capable of secreting and forming an elaborate reticular network within the LN. Single layer of FRCs enwrap extracellular matrix (ECM) that consists of a central core formed by 20–200 parallel bundles of fibrillar collagens (I and III) and intervening matrix of fibrils (14–16). These collagen bundles are surrounded by a layer of fibrillin-constituted microfibrils that are further ensheathed by a unique basement membrane-type structure (15, 16). In addition, stabilizing and cross-linking molecules such as fibromodulin, decorin, and lumican are also associated with the collagen fibers (17). FRCs also express other ECM component including ER-TR7 and common basement membrane component such as laminin and fibronectin (13). Integrin subunits and adhesion ligands such as intercellular adhesion molecule 1 (ICAM-I) and vascular cell adhesion molecule 1 are also found in FRCs (13). The three-dimensional tubular conduit system formed by FRCs extend the SCS throughout the T cell zone and form a contiguous lumen with fluid channels around the HEVs (18). Small lymph-borne molecules including chemokines and antigens from upstream periphery are transported within the core of FRC conduits from the SCS toward the HEVs. Molecules of high molecular mass (>70 kDa) cannot gain access to the conduit lumen and hence circumvent the lymphoid compartment and drained along the sinuses into the efferent lymphatic vessels (1, 4). Large particles including whole virus particles can also be captured by SCS macrophages and presented to migrating B cells in the underlying follicles (2, 4, 19).

In addition to acting as a key structural component in the LNs, FRCs are actively engaged in functional interactions with hematopoietic cells by forming conduits for antigens and inflammatory stimuli (1, 18), maintaining T cell survival (20), providing “tracks” and chemokines cue to guide cellular movement (21, 22), and supporting DC–T–B cell interactions during immune response (23) and peripheral tolerance (24–26). Disruption of FRC integrity and organization in the LNs during viral infection leads to profound loss of immunocompetence (27) strongly underscoring the roles of FRCs in maintaining proper immune response.

## Lymphatic Endothelial Cells

Lymphatic vessels are present in most tissues and are important for maintenance of fluid homeostasis, immune cells trafficking, and movement of soluble antigens (28). Lymph from upstream peripheral tissues first passes through the SCS, a space underneath the collagen-rich fibrous capsule that covers the LN. The floor of SCS is lined by LECs expressing lymphatic vessel endothelial hyaluronan receptor 1 (LYVE-1) and is interspersed with CD169^+^ macrophages and DCs. From there, lymph percolates through the highly branched medullary sinuses and blind-ended cortical sinuses before leaving the LNs *via* the efferent lymphatic vessel (6). Cortical LECs form the vessels and branch into the T cell zone and have been indicated to facilitate B and T cell egress (29–31). Medullary sinuses lined by LYVE-1^+^ endothelium are found at LN exit within the medulla. Recently, the markers to delineate the LECs located in the SCS, cortex, and medulla have been reported and include programmed death ligand 1 (PD-L1), ICAM-1, MadCAM-1, and lymphotoxin β receptor (32).

Research on LN LECs in the past decades has demonstrated that lymphatic vessels are not “inert conduits” but rather plastic structures that actively sense and respond to changes in the peripheral tissue environment. For example, inflammation induced by bacterial pathogen, immunization in the presence of complete Freund’s adjuvant, and contact sensitization have been shown to promote the growth of lymphatic vessels from preexisting ones, a process named lymphangiogenesis, in LNs (33–37). Furthermore, it becomes apparent that such lymphatic remodeling in LN can have important biological consequences including modulation of inflammation and adaptive immune responses (38–41). Indeed, a growing body of evidence is now demonstrating that LECs themselves can help shape adaptive immune responses through their interactions with key immune cells including DCs, macrophages, and lymphocytes. Owing to their migration through and within lymphatic vessels and their anatomical distribution in LNs, T cells frequently encounter LECs. This review focuses on the crosstalk between T cells and LECs in LNs and its immunological consequences.

## LN LECs Control T Cell Pool

### LECs Regulate T Cell Migration to, within, and out of LN

We will briefly discuss in this section how LECs attract and facilitate the trafficking of T cells from the periphery to LN and within the LN since this topic has been covered in depth in excellent reviews (42, 43). Although LECs have been shown to express a large number of chemokines that attract T cells (38), the role of CCL21 is the most established in the homing of naïve, memory, and T regulatory (Treg) T cells to LNs. The signaling induced by CCL21 binding to its receptor, CCR7, on the surface of migratory T cells is critical for T cell trafficking from the periphery to the LN as shown in mice deficient for CCR7 ligands (44). Then, LECs in the cortical sinuses regulate intranodal lymphocyte trafficking by collecting lymphocytes for further transit to medullary sinuses (45). Moreover, lymphocytes can frequently move from the lymphatic sinuses back to the LN parenchyma (45). In line with these findings, it was reported that lymph-borne lymphocytes are passively transported into the peripheral medullary sinuses. Subsequently, they enter the LN parenchyma independently of CCR7 signals by migrating into adjacent peripheral medullary cords (46).

Medullary sinuses are directly connected to the efferent lymphatic vessel and have been proposed in addition to cortical sinuses as exit routes for the egress of lymphocytes from LNs (29, 30, 45). The molecular mechanisms of lymphocyte egress mediated by LECs remain elusive, and further investigations will be needed to explain how medullary sinuses can serve as both entry and egress structures for T cells. Most work on T cell egress has focused on mechanisms that lymphocytes uses to reach efferent lymphatic vessels and has identified sphingosine-1-phosphate (S1P)/S1P1 as a critical signal axis in promoting T cell egress (47). S1P levels are low in LN parenchyma but high in lymph fluid, thus creating a gradient. This S1P gradient guides T cells exhibiting decreased CCR7-retention signals from LN parenchyma into medullary and cortical sinuses and ultimately facilitates T cell egress (48). Notably, S1P in cortical sinuses and efferent lymph has been shown to be produced by LYVE-1^+^ LECs. Mice lacking specifically S1P kinase, the enzyme responsible for S1P synthesis, in LECs show compromised T cell egress (49). It is well established that local immune responses and inflammation are accompanied by alterations in the trafficking of lymphocytes through LNs. Specifically, the entry of lymphocytes into LNs increased, whereas their egress into efferent lymph is temporarily inhibited for few hours to days, depending on the nature of the stimulus (50–52). Few years ago, we reported that inflammation in LN, as it evolves from early to late phases, can induce a biphasic remodeling of lymphatic network, with the SCSs being expanded first, followed by the cortical and medullary sinuses. We showed that the early expansion of SCSs enhances the migration of DCs from the periphery, whereas the preferential expansion of cortical and medullary sinuses at later stages of inflammation supports the restoration of lymphocyte egress to steady-state levels (53).

### LN LECs Support the Survival of T Cells

Several emerging evidence indicates that LECs may not only regulate the homeostasis of T cells in LNs through the modulation of their migration but also their survival. Interleukin (IL)-7 binds to IL-7Rα chain in combination with the common-γ chain and is essential for T lymphocyte homeostasis within the secondary lymphoid organs. IL-7 expression *in vivo*, which appears to limit the size of the lymphocyte pool, was thought to be regulated by IL-7 receptor α (IL-7Rα)-mediated consumption rather than the rate of IL-7 expression (54, 55). However, this concept has been recently challenged by a study showing that IL-7 expression can be induced in the liver in response to Toll-like receptor signaling and can directly control T cell responses (56). In line with this latter study, an earlier report by the same group demonstrated that excessive IL-6 expression increases IL-7 expression, which in turn was associated with the development of autoimmune reaction (57). These studies underscore that production of IL-7 by non-hematopoietic cells is tightly and dynamically regulated. In LNs, IL-7 provides antiapoptotic and proliferative signals to naïve and memory T cells (58–61). Although FRCs have been shown to be a major producer of IL-7 in LNs (20), it appears now evident that LECs are also an important source of IL-7 in murine and human LNs (62, 63). Interestingly, during inflammation-induced LN remodeling that influences intranodal lymphocyte dynamics, IL-7-expressing cortical sinus LECs have been shown to be essential for LN remodeling (63). In line with the role of IL-7 in maintaining memory T cells, a recent study revealed that LECs in lungs from mouse and humans can support the survival of memory T-helper cells through the production of IL-7 and IL-33 during allergic airway inflammation (64). IL-33 is a pro-inflammatory cytokine that initiates chronic inflammation in the lung, and its receptor is highly expressed on memory Th2 cells. IL-33 has been shown to directly induce memory Th2 cells to produce IL-5 and induces eosinophilic inflammation. Although this study focuses on lung LECs, it raises the possibility that LECs through the production of diverse cytokines may control the survival of pathogenic T cells during chronic inflammation, which in turn may have serious pathological consequences. Furthermore, the fact that IL-7 has been shown to mediate the transition from effector into memory T cells (65, 66) may also suggest the potential implication of LECs in shaping T cell differentiation in LNs during immune response.

## LN LECs Regulate T Cell Activation

### LN LECs Function as APCs for Peripheral T Cell Tolerance

Peripheral immune tolerance is generally ascribed to quiescent tissue-resident DCs cross-presentation of tissue-associated antigens to self-reactive T cells that have escaped thymic negative selection (67). More recently, accumulating evidence demonstrates that direct presentation of self-antigens by LN stromal cell subsets including FRCs and LECs can also mediate peripheral tolerance (25, 26, 68). Among LN stromal cell populations, LECs are likely the first cells that are in direct contact with the antigens, danger signals, and immune cells that carry peripheral blueprint to the draining LN. LECs express MHC class I (68–70) and MHC class II (41, 71, 72) and are capable of inducing T cell tolerance directly and suppressing DC-mediated T cell activation. In addition, T cell activation is also affected by the cytokine environment and relative balance between costimulatory and inhibitory signals from the APCs (41, 71, 73–75).

There are several potential pathways by which LECs can induce T cell tolerance. For instance, LN LECs express multiple peripheral tissue antigens (PTAs) (25, 69). In steady state, LECs lack costimulatory molecules such as CD80, CD86, or 4-1BBL that normally drive immunogenic T cell response. Instead, high expression of PD-L1 on LECs and engagement with its receptor on T cells predispose them to promote peripheral T cell tolerance (41). In a model of LEC-induced tolerance of melanocytes differentiation protein tyrosinase-specific CD8^+^ T cells, lack of stimulation through 4-1BB led to rapid and increased expression level of PD-1. Signaling through PD-1 inhibits upregulation of IL-2R on CD8^+^ T cells, culminating in apoptotic death associated with the loss of IL-2 prosurvival signaling (41). On the other hand, rescue of tyrosinase-specific CD8^+^ T cells by interfering PD-1 signaling or providing costimulatory signals gain effector function and induce autoimmune vitiligo, demonstrating that LECs are important and specialized APCs for peripheral T cell tolerance (41). This latter finding is in line with the observation in severe enteric autoimmunity that loss of PD-1/PD L1 inhibitory pathway blocks CD8^+^ T cell tolerance to intestinal self-antigens (76). It is worth to note that tyrosinase and PD-L1 are expressed at higher levels in LN LECs as opposed to LECs in periphery (diaphragm or colon), indicating that the LN microenvironment endows LN LECs with tolerogenic properties not found in tissue LECs (32). Given that LECs express various PTAs, dysregulation of LEC-associated tolerance is likely expected to contribute to the development of several autoimmune disorders.

In addition to transcriptionally expressed PTAs, LN LECs have also been shown to scavenge and cross-present exogenous antigen to naïve CD8^+^ T cells in the model of B16 F10 melanoma expressing the foreign antigen ovalbumin (OVA) and overexpressing vascular endothelial growth factor (VEGF)-C (70). VEGF-C-induced LN lymphangiogenesis suppresses anti-tumor immunity by local deletion of OVA-specific CD8^+^ T cells, which in turn drives disease progression and metastatic outgrowth. Similar observation was also reported under homeostatic conditions whereby intradermal injection of fluorescently labeled OVA protein was engulfed by LN LECs, processed, and presented on MHC class I to cognate CD8^+^ T cells in a TAP1-dependent manner (77). Such T cell/LEC interaction was shown to lead to decreased cytokine production and increased expression of Annexin V and exhaustion markers (PD-1, CD80, and CTLA-4) *in vitro* (77). These experimental findings suggest that regardless of the source of antigen (exogenous or endogenous), constitutive expression of inhibitory molecules and lack of costimulatory molecules on LECs will predominantly induce peripheral tolerance.

Furthermore, LECs express intermediate levels of MHC class II molecules suggesting that they might also tolerize CD4^+^ T cells (41, 71). MHC class II on LECs has shown to be either acquired from the DCs or endogenously expressed (24, 72). Rouhani et al. employed transgenic systems where antigens β-galactosidase (β-gal) and hemagglutinin (HA) were conditionally expressed in LECs under the control of Prox-1 and LYVE-1 promoters (72). Both CD8^+^ and CD4^+^ T cell receptors are available in these models and hence allowing comparative evaluation of the ability of LECs to drive tolerance to epitopes from the same protein presented by either MHC class I or MHC class II molecules. The authors demonstrated that PTA β-gal and HA epitopes on MHC class I were directly presented to CD8^+^ T cells, whereas these epitopes on MHC class II molecules were not presented to CD4^+^ T cells both *in vivo* and *in vitro*. Instead, these antigens were transferred to DC and then presented to CD4^+^ T cell to induce anergy. Therefore, LECs serve as a reservoir and repertoire of PTAs in the LN that may be acquired by DCs to induce tolerogenic CD4^+^ T cells. Similarly, Dubrot et al. showed that LECs acquire peptide: MHC class II complexes from DCs (24). However, in contrast to Rouhani et al., these complexes were not observed to be transferred back to LECs in sufficient quantities to induce CD4^+^ T cells recognition and subsequent antigen-specific T cells apoptosis.

### LN LECs Modulate DC Functions

LECs may also regulate T cell activation indirectly by modulating antigen-presenting functions of DCs. Under steady state, immature DCs typically capture autoantigens from apoptotic cells, migrate to LNs, and promote T cell tolerance (78–80). Exposure of DCs to danger signals during inflammation or infection increases the expression of MHC class II molecules, costimulatory molecules, and cytokine that ultimately can trigger immunity and prevent tolerance. LECs have been shown to attenuate T cell response by suppressing DC maturation (73, 74, 81). Direct contact of immature DCs with an inflamed, TNF-α-stimulated LECs decreases expression of CD86 on DCs, dampening their ability to stimulate T cell proliferation (81). This interaction was mediated by the binding of ICAM-1 on LECs to Mac-1 on DCs and was observed in the absence of PAMPs (81). LECs also restrain T cell proliferation through upregulation of nitric oxide synthase-2 and production of NO in response to interferon (IFN)-γ and TNF-α released from activated T cells (73). Furthermore, IFN- γ-stimulated cultured human LN LEC produces inhibitory indoleamine 2,3 dioxygenase that in turn impairs CD4^+^ T cell proliferation (74). Interestingly, in different contexts such as viral challenge and subunit vaccination, viral antigens are captured and archived in LECs and subsequently transferred to DCs for the maintenance of memory T cells and enhancement of protective immunity (82). Therefore, crosstalk between LECs and DCs within the LN can either drive tolerogenic or immunogenic responses depending on the antigenic stimuli, immune cells encountered, and the type of inflammatory challenges.

### LN LECs Archive Antigens

Several studies have reported that persistence of virally associated antigens after acute infection and subsequent viral clearance or so-called the reservoir of antigens was localized within the LNs draining the site of initial infection (82–86). A recent report demonstrated that LN LECs retain persisting antigens for weeks after vaccination (82). This antigen archiving was dependent on the induction of LN lymphatic proliferation. However, LECs did not present directly the archived antigen to T cells but instead required hematopoietic APCs. The number and percentage of CD8^+^ T cell-producing IFN-γ and IL-2 were significantly increased when antigen was retained in LECs. Notably, we previously reported that LN lymphangiogenesis persists during prolonged inflammation (53). Thus, it is plausible that the persistence of an expanded LN lymphatic network after viral infection or vaccination may allow the long-term storage of viral antigens. As a consequence, ongoing antigen presentation and recognition by memory T cells may lead to selective enrichment of virus-specific memory T cells in the draining LN even after the clearance of the infectious agent. This enriched population of antigen-specific T cells may provide more rapid effector responses in the periphery and better control of secondary infections.

## T Cells Control LEC Growth upon Inflammation

Because lymphangiogenesis in LN has been shown to have diverse functional consequences on inflammation and immune responses depending on the context and timeframe of its occurrence (39, 40), this process is expected to be highly regulated. Indeed, a large number of studies have identified cellular and molecular mechanisms promoting the growth of lymphatic vessels. In contrast, little knowledge is currently available on pathways counter-regulating lymphangiogenesis. Both non-immune and immune cells have been described to orchestrate the expansion of lymphatic vessel network within LN. Interestingly, among immune cells, B and T cells have been shown to have opposite effects, namely, B cells support inflammatory lymphangiogenesis in LNs, whereas T cells have antilymphangiogenic effects. The first evidence supporting a role for T cells as negative regulators of LEC growth arises from a mouse study in which T cells were ablated using athymic mice (37). This antilymphangiogenic effect of T cells in the athymic mice was restored by the adoptive transfer of CD4^+^ or CD8^+^ T cells. This study suggests that both CD4^+^ and CD8^+^ T cells may harbor an antilymphangiogenic property. Other studies in different mouse models of inflammatory lymphangiogenesis have further confirmed the regulatory function of CD4^+^ T cells on LEC growth (34, 87). In the model of LN lymphangiogenesis induced by bacterial lipopolysaccharide, the authors demonstrated that the secretion of IFN-γ by T cells accounts for the inhibitory effect of T cells on LN lymphangiogenesis (37). Moreover, in line with an earlier study (88), they showed using *in vitro* cultured LECs that IFN-γ can act directly on LECs and affect their proliferation and survival (see Table 1) (37, 88).

**Table 1:** **Cytokines regulating lymphatic endothelial cell (LEC) growth**.

Cytokine	Mechanism	Model system	Reference
Interferon (IFN)-γ	Inhibits proliferation and migration	Cultured pig thoracic duct LEC	(88)
	
	Increases apoptosis		
	
	Inhibits lymph node (LN) lymphangiogenesis	LPS-induced LN lymphangiogenesis in mouse; Lewis Lung carcinoma cell implantation in mouse	(37)
	
	Inhibits proliferation and tube formation; downregulates Prox-1 LYVE-1and podoplanin expression	Cultured murine thoracic duct LEC	

Interleukin (IL)-4/IL-13	Inhibits LN lymphangiogenesis	CFA/ovalbumin-induced LN lymphangiogenesis	(34)
	
	Inhibits corneal lymphangiogenesis	Mouse model of suture-induced corneal neovascularization	(89)
	
	Inhibits proliferation, tube formation and migration; increases apoptosis	Cultured human dermal LEC	
	
	Inhibits lung and trachea lymphangiogenesis	Mouse model of allergen-induced asthma	(90)
	
	Inhibits proliferation, tube formation and downregulates Prox-1 and LYVE-1 expression	Cultured murine LN LEC and human dermal LECs	
	
	Increases skin lymphangiogenesis and promotes recruitment of macrophages and vascular endothelial growth factor (VEGF)-C expression	K14-IL-4 transgenic mouse	(91)

IL-17	Increases corneal lymphangiogenesis *via* VEGFR-3/VEGF-C/-D pathway	Mouse model of cornea micropocket and Th17-dominant autoimmune dry eye disease	(92)
	
	Increases proliferation and tube formation *via* VEGFR-3-dependent pathway	Cultured human dermal LECs	

IL-10	Increases lymphangiogenesis and promotes VEGF-C production by macrophages	Mouse model of suture-induced corneal neovascularization	(93)
	
	No direct effect on LEC	Cultured human dermal LECs	

TGF-β	Inhibits lymphangiogenesis	Mouse model of chronic peritonitis	(94)
	
	Inhibits proliferation, tube formation, and migration; downregulates Prox-1 and LYVE-1 expression	Cultured human dermal LEC	
	
	Inhibits proliferation and tube formation	Cultured human dermal LEC	(95)
	
	Independent of VEGF-C/-D		
	
	Inhibits lymphangiogenesis	Mouse lymphedema model	(96)
	
	Promotes lymphangiogenesis and upregulates VEGF-C expression	Rat model of unilateral ureteral obstruction	(97)
	
	Promotes lymphangiogenesis and upregulates VEGF-C expression	Rat model of peritoneal fibrosis	(98)
	
	Enhances branching and sprouting of lymphatic network in embryonic skin	E13.5–15.5 mouse embryos	(99)
	
	Attenuates LEC proliferation	Cultured human dermal microvascular LECs	

These two latter studies provided the first evidence for a role of cytokines in controlling the expansion of lymphatic vessels. Since then, this notion has been further validated by several recent studies reporting the effect of other cytokines including IL-10, IL-17, TGF-β, and IL-4/IL-13 on LEC growth *in vitro* and/or in diverse models of inflammatory or *de novo* lymphangiogenesis induced in LN or other tissues (Table 1). From these studies, it becomes apparent that (i) cytokines are not always antilymphangiogenic; (ii) one given cytokine may have prolymphangiogenic or antilymphangiogenic properties depending on the context in which lymphatic growth occurs; and (iii) modulation of lymphatic proliferation, survival, and migration by cytokines can be mediated by a direct effect on LECs or indirectly by controlling the expression of lymphangiogenic factors such as VEGF-A, -C, and -D. Interestingly, all these cytokines can be secreted by different CD4^+^ T subsets including Th1, Th2, Th17, and Treg cells raising the possibility that different T cell subsets recruited to LN may affect LEC growth. Although this notion is indirectly supported by the studies cited in Table 1 and a recent study reporting the effect of Treg on lymphatic transport in a mouse model of lymphedema (100), direct evidence for a role of these T cell subsets and their cytokines in controlling LN lymphangiogenesis is lacking.

## Implications of LEC Immunomodulatory Properties in Cancer Progression

**Figure 1:**
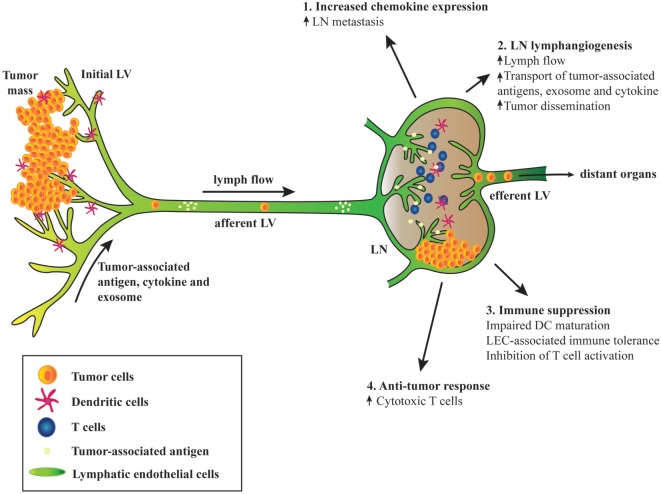
**Schematic diagram depicting the involvement of tumor-associated lymphatic endothelial cell (LEC) in cancer**. (1) Tumor-associated upregulation of chemokine expression in lymph node (LN) LECs mediates metastasis of tumor cells expressing the cognate chemokine receptors. (2) Tumor-associated factors, cytokines, and exosome draining from the upstream tumors and afferent lymphatic induce LN lymphangiogenesis, leading to increased lymph flow, transport of tumor-derived factors, and enhanced tumor cell dissemination. (3) Tumor-associated LECs can suppress immunity and promote tolerance. Interaction between LN LECs and dendritic cells (DCs) *via* intercellular adhesion molecule 1 and Mac-1 inhibits DC maturation and hence limiting effective T cell activation. Tumor antigen presentation to naïve CD8^+^ T cells by LN LECs induces dysfunctional T cell activation and tolerance due to expression of inhibitory receptor programmed death ligand 1 and lack of costimulatory molecules on LEC surface. LECs activated by T cell-derived pro-inflammatory cytokines produce factors such as NO and indoleamine 2,3 dioxygenase that inhibit T cell proliferation. (4) Robust CD8^+^ T cells priming occurs in tumor-draining LN. Although tolerogenic LN microenvironment may dominate and sustain immune suppression, immune checkpoint blockades can reverse T cell exhaustion and increase effector T cell activities that may lead to tumor regression.

The ever-growing research on tumor biology, immunology, and lymphatic biology has recently highlighted the multifaceted roles of lymphatic vessels in shaping tumor immunity and in cancer progression. One of the cardinal functions of lymphatic vessel is to transport components of the local tissue containing interstitial solutes, cytokines, growth factors, and immune cells to the downstream LN for the maintenance of tissue fluid homeostasis and peripheral immune tolerance. Tumor cells can “hijack” the lymphatic and induce the expansion of lymphatic vessels for their dissemination, colonization, and the formation of metastasis in the tumor-draining LNs (101, 102) (Figure 1). *Via* the lymphatic route, tumor cells can also modify the microenvironment of the metastatic organs from the distal sites before their arrival—referred to premetastatic niche. LN lymphangiogenesis preceding metastasis is an important mechanism and is associated with cancer progression (103–106).

Moreover, LN LECs express several chemokines that can attract cancer cells expressing the cognate chemokine receptors. For instance, constitutive CCL21 expression by LEC can serve as a guide for CCR7-expressing breast cancer and melanoma cells invading the LNs (107). Overexpression of CCR7 in melanoma has been shown to promote LN metastasis in mice (108), and CCR7 expression in human cancer samples correlates positively with LN metastasis (109–111). Upregulation of CXCL12 expression has been reported to enhance LN metastasis of CXCR4^+^ tumor cells (112). CCL1 is another chemokine produced by the SCS LEC, which has been shown to control CCR8^+^ tumor cell entry and subsequent migration and colonization in the LN cortex (113). Blocking of CCL1-CCR8 signaling results in the arrest of tumor cells at the junction of the afferent lymphatic vessels and the LN.

As discussed earlier, LN LECs can profoundly affect T cell survival, fate, and activation that can be of significant importance in tumor immune responses (Figure 1). The primary tumor is connected to the downstream afferent lymphatic vessel and draining LNs, and this connection may allow the entry of tumor-derived factors to the draining LNs and consequently may alter regional immune responses. Such alterations were reported to occur even before LN metastasis (114). Moreover, owing to the lack of costimulatory molecules expression and high levels of inhibitory ligand PD-L1 on LN LECs, lymphatic antigen presentation *via* MHC-I can induce deletional tolerance, a mechanism by which tumor cells may evade host immunity (41, 69, 70). VEGF-C-induced LN lymphangiogenesis can further promote immune tolerance in B16 melanoma-implanted mouse model (70). However, these studies suggest that manipulating LEC-associated tolerance or cancer dissemination may create opportunities for a new generation of antitumor immunotherapy. Importantly, cancer immunotherapies targeting the immune checkpoints, PD-1 and PD-L1, are revolutionizing current cancer treatments (115, 116). In humans, anti-PD-1 antibodies that target tumor-specific T cells (117–119) and anti-PD-L1 antibodies that bind to ligand expressed by the tumor and intratumor immune cells (120, 121) show promising clinical benefits. One can speculate that targeting this PD-1/PD-L1 immune checkpoint *via* systemic administration may also interrupt the tolerogenic signaling pathway between LN LECs and CD8^+^ T cells. Perhaps, a more LN-specific delivery of these blocking antibodies or other anticancer vaccine may lead to a greater impact on antitumor immune responses (122).

Although LN LECs may contribute to immune suppressive environment within the tumor-draining LNs (whether by direct interaction with CD8^+^ T cells or by draining the immunosuppressive cytokines from the upstream tumors), their roles in tumor immune surveillance cannot be neglected (Figure 1). Indeed, circulating tumor-specific T cells in metastatic melanoma patients are functional although those isolated from tumor-draining LNs exhibit exhausted characteristics (decreased IFN-γ and increased CTLA-4 and LAG-3 expression) (123). Interestingly, co-administration of anti-CTLA-4 and PD-1 antibodies reverses T cell exhaustion by increasing effector T cell activity and cytokine production and hence augmenting tumor inhibition (124). Tumor immunity was examined in the context of impaired lymphatic function using a *kCYC* transgenic mouse model expressing Kaposi’s sarcoma-associated herpes virus latent-cycle gene, *k-cyclin*, and under the control of VEGFR-3 promoter (101). In this model, antigen-presenting ability of DCs and cytotoxicity of CD8^+^ T cells isolated from the draining LNs of *kCYC* mice were attenuated. Furthermore, adoptive transfer of CD8^+^ T cells derived from *kCYC* mice to naïve WT mice show impaired antitumor function (101). In another model of dermal lymphatic insufficiency (K14-VEGFR3-Ig mice), implanted melanoma grew robustly and exhibited marked reduction in leukocyte infiltration compared with those implanted in control mice, suggesting that lymphatic vessels are essential for the generation of tumor immune responses (125). In addition, we showed in a spontaneous mouse model of uveal melanoma that early resection of TDLNs promotes primary tumor growth, cancer cell dissemination, and metastasis (102). Even though we did not examine the role of immune responses in the absence of tumor-draining LNs, it is plausible that uncontrollable growth of primary tumor may be due to the lack of antitumor immunity since the depletion of CD8^+^ T cells accelerates tumor growth and dissemination in the same model (126). These reports strongly indicate that functional lymphatic and presence of tumor-draining LNs are required for cancer immune surveillance. To further support this, current cancer immunotherapies targeting the immune checkpoints have demonstrated and supported the evidence that antitumor immunity exists even in the most advanced stages of cancer (116, 127–129).

## Concluding Remarks

The ever-growing research on lymphatic biology has clearly identified LECs as key players in regulating adaptive immunity particularly by affecting T cell functions. However, the dynamics of T cells/LECs interactions and their immunological consequences in the context of cancer need to be further delineated. LN LECs are intricately affected by peripheral tumor, tumor-associated factors, and immune cells that in turn enhance tumor cell dissemination and drive the balance between host immunity and tolerance. Hence, LN LECs may represent a potential therapeutic target in addition to immunotherapy strategies for cancer progression and metastasis. Although tumor-associated LN lymphangiogenesis can contribute to tumor dissemination and increased immune tolerance, LN LECs are also important for the communication between tumors and immune cells to mount antitumor immune responses. For these reasons, combined research on immunology, lymphatic, and tumor biology is essential to further elucidate the immunological roles of LN LECs in cancer and their impact on disease progression.

## Author Contributions

All the authors listed have written the manuscript and VA approved it for publication.

## Conflict of Interest Statement

The authors declare that the research was conducted in the absence of any commercial or financial relationships that could be construed as a potential conflict of interest.
